# Managing COVID-19 With a Clinical Decision Support Tool in a Community Health Network: Algorithm Development and Validation

**DOI:** 10.2196/22033

**Published:** 2020-08-24

**Authors:** Michael P McRae, Isaac P Dapkins, Iman Sharif, Judd Anderman, David Fenyo, Odai Sinokrot, Stella K Kang, Nicolaos J Christodoulides, Deniz Vurmaz, Glennon W Simmons, Timothy M Alcorn, Marco J Daoura, Stu Gisburne, David Zar, John T McDevitt

**Affiliations:** 1 Department of Biomaterials, Bioengineering Institute New York University College of Dentistry New York, NY United States; 2 Department of Population Health and Internal Medicine Family Health Centers at NYU Langone New York University School of Medicine New York, NY United States; 3 Departments of Pediatrics and Population Health Family Health Centers at NYU Langone New York University School of Medicine New York, NY United States; 4 Family Health Centers at NYU Langone New York, NY United States; 5 Department of Biochemistry and Molecular Pharmacology New York University School of Medicine New York, NY United States; 6 Department of Medicine New York University School of Medicine New York, NY United States; 7 Department of Radiology New York University School of Medicine New York, NY United States; 8 Department of Population Health New York University School of Medicine New York, NY United States; 9 Department of Chemical and Biomolecular Engineering NYU Tandon School of Engineering New York University New York, NY United States; 10 Latham BioPharm Group Cambridge, MA United States; 11 OraLiva Naples, FL United States

**Keywords:** COVID-19, coronavirus, clinical decision support system, point of care, mobile app, disease severity, biomarkers, artificial intelligence, app, family health center

## Abstract

**Background:**

The coronavirus disease (COVID-19) pandemic has resulted in significant morbidity and mortality; large numbers of patients require intensive care, which is placing strain on health care systems worldwide. There is an urgent need for a COVID-19 disease severity assessment that can assist in patient triage and resource allocation for patients at risk for severe disease.

**Objective:**

The goal of this study was to develop, validate, and scale a clinical decision support system and mobile app to assist in COVID-19 severity assessment, management, and care.

**Methods:**

Model training data from 701 patients with COVID-19 were collected across practices within the Family Health Centers network at New York University Langone Health. A two-tiered model was developed. Tier 1 uses easily available, nonlaboratory data to help determine whether biomarker-based testing and/or hospitalization is necessary. Tier 2 predicts the probability of mortality using biomarker measurements (C-reactive protein, procalcitonin, D-dimer) and age. Both the Tier 1 and Tier 2 models were validated using two external datasets from hospitals in Wuhan, China, comprising 160 and 375 patients, respectively.

**Results:**

All biomarkers were measured at significantly higher levels in patients who died vs those who were not hospitalized or discharged (*P*<.001). The Tier 1 and Tier 2 internal validations had areas under the curve (AUCs) of 0.79 (95% CI 0.74-0.84) and 0.95 (95% CI 0.92-0.98), respectively. The Tier 1 and Tier 2 external validations had AUCs of 0.79 (95% CI 0.74-0.84) and 0.97 (95% CI 0.95-0.99), respectively.

**Conclusions:**

Our results demonstrate the validity of the clinical decision support system and mobile app, which are now ready to assist health care providers in making evidence-based decisions when managing COVID-19 patient care. The deployment of these new capabilities has potential for immediate impact in community clinics and sites, where application of these tools could lead to improvements in patient outcomes and cost containment.

## Introduction

Coronavirus disease (COVID-19) was first reported in Wuhan, Hubei, China, in December 2019 [1], and it was declared a pandemic by the World Health Organization (WHO) [2] soon thereafter. As of June 15, 2020, about 8 million cases have been confirmed, with approximately 435,000 deaths from the disease worldwide [3]. The COVID-19 crisis has exposed critical gaps in diagnostic testing and population-level surveillance [4]. With hospitalization rates of 20% to 31% and intensive care unit (ICU) admission rates of 5% to 12% [5], surges of patients are requiring care, which has overwhelmed local health care systems and depleted reserves of medical resources.

Physicians are tasked with evaluating large amounts of rapidly changing patient data and making critical decisions in a short amount of time. Well-designed clinical decision support systems (CDSSs) deliver pertinent knowledge and individualized patient information to health care providers to enhance medical decisions [6]. These systems may rely on surveys of similar cases, while others may use a “black box” approach [7]. Traditional scores such as Sepsis-related Organ Failure Assessment (SOFA) [8-10] and Acute Physiology and Chronic Health Evaluation (APACHE) II [11,12] are commonly used in hospitals for determining disease severity and mortality, whereas clinical decision management systems, such as electronic ICU (eICU), enable systematic collection of comprehensive data [13]. However, CDSSs that use conventional variables, such as demographics, symptoms, and medical history, often do not reach their full diagnostic potential [14]. There is a compelling need for a COVID-19 disease severity assessment to help prioritize care for patients at elevated risk of mortality and manage low-risk patients in outpatient settings or at home through self-quarantine.

Several scoring systems for COVID-19 severity have been developed or adapted from existing tools, such as the Brescia-COVID Respiratory Severity Scale [15], African Federation for Emergency Medicine COVID-19 Severity Scoring Tool [16], Berlin Criteria for Acute Respiratory Distress Syndrome [17,18], and Epic Deterioration Index [19]. However, these tools have either not yet been externally validated in peer-reviewed publications or were not developed specifically for COVID-19 patient populations. Recently, we developed an integrated point-of-care COVID-19 Severity Score and CDSS that combines multiplex biomarker measurements and risk factors in a statistical learning algorithm to predict mortality with excellent diagnostic accuracy [20]. The COVID-19 Severity Score was trained and evaluated using data from 160 hospitalized COVID-19 patients from Wuhan, China. The COVID-19 Severity Score was significantly higher for patients who died than for patients who were discharged, with median scores of 59 (IQR 40-83) and 9 (IQR 6-17), respectively, and an area under the curve (AUC) of 0.94 (95% CI 0.89-0.99).

COVID-19 has caused and continues to cause significant morbidity and mortality globally. A validated tool to assess and quantify viral sepsis severity and patient mortality risk would address the urgent need for disease severity categorization. Toward the goal of improving prognostic judgement and outcomes, we assembled a multidisciplinary team representing stakeholders from technology, machine learning, engineering, primary care, and in vitro diagnostic testing to develop a COVID-19 disease severity test. The unfolding novel COVID-19 pandemic has greatly illuminated the important role of community health centers in providing safe and effective patient care. The Family Health Centers (FHC) at New York University (NYU) Langone is a large Federally Qualified Health Center; it provides comprehensive primary and preventive health care to a diverse population of patients across the New York City metropolitan area and is well-positioned to improve survival by fast-tracking hospitalization of patients at high risk of severe disease. This study describes a clinical decision support tool for COVID-19 disease severity developed using recent data from the FHC and externally validated using data from two recent studies from hospitals in Wuhan, China. We describe a practical and efficient tiered approach that involves a model with nonlaboratory inputs (Tier 1), a model with biomarkers commonly measured in ambulatory settings (Tier 2), and a mobile app to deliver and scale these tools. The deployment of these new capabilities has potential for immediate clinical impact in community clinics, where these tools could lead to improvements in patient outcomes and prognostic judgment.

## Methods

### Patient Data

Data from 701 patients with COVID-19 were collected across 9 clinics and hospitals within the FHC network at NYU Langone, one of the largest Federally Qualified Health Center networks in the United States. All patients had detectable severe acute respiratory syndrome coronavirus 2 (SARS-CoV-2) infection as evidenced by polymerase chain reaction testing. The following outcomes were recorded: not hospitalized, discharged, ventilated, and deceased. The data that support the Tier 1 Outpatient Model and Tier 2 Biomarker Model development are available from the authors upon reasonable request and with permission of FHC at NYU Langone. Validation data for the Tier 1 Outpatient Model were derived from a study of 160 hospitalized patients with COVID-19 from Zhongnan Hospital of Wuhan University. The data that support validation of the Tier 1 Outpatient Model are available from the authors upon reasonable request and with permission of Zhongnan Hospital of Wuhan University. Validation data for the Tier 2 Biomarker Model were derived from a study of 375 hospitalized patients with COVID-19 from Tongji Hospital in Wuhan, China. The data that support the validation of the Tier 2 Biomarker Model are available as Supplementary Data in a publication by Yan et al [21].

### Clinical Decision Support Tool

This study describes the development of a two-tiered CDSS for the assessment of COVID-19 disease severity using similar methods to those described previously [20,22]. The Tier 1 Outpatient Model uses nonlaboratory data that are readily available prior to laboratory measurements and is intended to help determine whether Tier 2 biomarker-based testing and/or hospitalization are necessary. Here, a lasso logistic regression model was trained to distinguish between patients who were not hospitalized or who were hospitalized and discharged home without need for ventilation vs patients who were ventilated or died. Patients who were still hospitalized when the data were compiled were excluded. The following predictors were considered in model training: age, gender, BMI, systolic blood pressure, temperature, symptoms (cough, fever, or shortness of breath), known cardiovascular comorbidities (patient problem list includes one or more of cerebrovascular disease, heart failure, ischemic heart disease, myocardial infarction, peripheral vascular disease, and hypertension), pulmonary comorbidities (asthma and chronic obstructive pulmonary disease), and diabetes.

The Tier 2 Biomarker Model predicts disease severity using biomarker measurements and patient characteristics. A lasso logistic regression model was trained to distinguish patients who died versus patients who were either never hospitalized or discharged home. Patients who were ventilated or still hospitalized when the data were compiled were excluded. The following predictors were considered in model training: age, gender, comorbidities, C-reactive protein (CRP), cardiac troponin I (cTnI), D-dimer, procalcitonin (PCT), and N-terminal fragment of the prohormone brain natriuretic peptide (NT-proBNP). Predictors that were not relevant to the model (ie, coefficients equal to zero) were removed. Laboratory measurements across all time points were log-transformed. Patients with no measurements for the aforementioned biomarkers were excluded. Biomarker values below the limits of detection were set to the minimum measured value divided by the square root of 2.

### Model Development and Statistical Analysis

Both Tier 1 and Tier 2 models were developed using the same procedure. All continuous predictors were standardized with a mean of 0 and a variance of 1. Missing data were imputed using the multivariate imputation by the chained equations algorithm in the statistical software R (R Project) [23]. Predictive mean matching and logistic regression imputation models were used to generate 10 imputations for continuous and categorical predictors, respectively. Samples in the training and test sets were partitioned using stratified 5-fold cross-validation to preserve the relative proportions of outcomes in each fold. Model training and selection were performed on each of the 10 imputation datasets for 10 Monte Carlo repetitions and optimized for the penalty parameter corresponding to one standard error above the minimum deviance for additional shrinkage. After the initial training, only predictors with nonzero regression coefficients were retained, and the model was retrained with a reduced number of predictors. The training process was repeated until all predictors yielded nonzero coefficients. Model performance was documented in terms of the mean (95% CI) of the AUC, sensitivity, specificity, positive predictive value (PPV), and negative predictive value (NPV). Median (IQR) cross-validated COVID-19 scores were compared across disease outcomes. The COVID-19 scores for both models and biomarker measurements were compared using the Wilcoxon rank sum test. Normally distributed predictors were compared using an independent *t* test. Proportions were compared using the chi-squared test [24,25]. Two-sided tests were considered statistically significant for *P*<.05.

### External Validation

We externally validated the Tier 1 Outpatient Model using data from a study of 160 hospitalized patients with COVID-19 from Zhongnan Hospital of Wuhan University. Only patients with complete information (age, systolic blood pressure, gender, diabetes, and cardiovascular comorbidities) were included. The model performance was documented in terms of AUC, sensitivity, specificity, PPV, and NPV. Results were presented in a scatter/box plot of COVID-19 outpatient scores for patients who were discharged and those who died.

Similarly, we externally validated the Tier 2 Biomarker Model using data from a study of 375 hospitalized patients with COVID-19 from Tongji Hospital in Wuhan, China, collected between January 10 and February 18, 2020 [21]. While most patients had multiple lab measurements over time, the first available lab value for each biomarker was used to validate the model to maximize lead time. Patients with one or more missing predictor values were excluded. Model performance was documented in terms of AUC, sensitivity, specificity, PPV, and NPV. Results were presented in a scatter/box plot of COVID-19 Biomarker Scores for patients who were discharged and who died.

To demonstrate how the COVID-19 Biomarker Score could be used to track changes in disease severity over time, the model was evaluated based on time series biomarker data. Because the lab measurements were reported asynchronously, the model was reevaluated every time a new biomarker measurement became available. Time series plots of the COVID-19 Biomarker Score were generated for each patient.

## Results

This study describes the development of a 2-tiered CDSS to assess COVID-19 disease severity using similar methods to those described previously [20,22]. The Tier 1 Outpatient Model uses nonlaboratory data that are readily available prior to laboratory measurements and is intended to help determine whether Tier 2 biomarker-based testing and/or hospitalization are warranted. The Tier 2 Biomarker Model predicts disease severity using biomarker measurements and patient characteristics.

**Figure 1:**
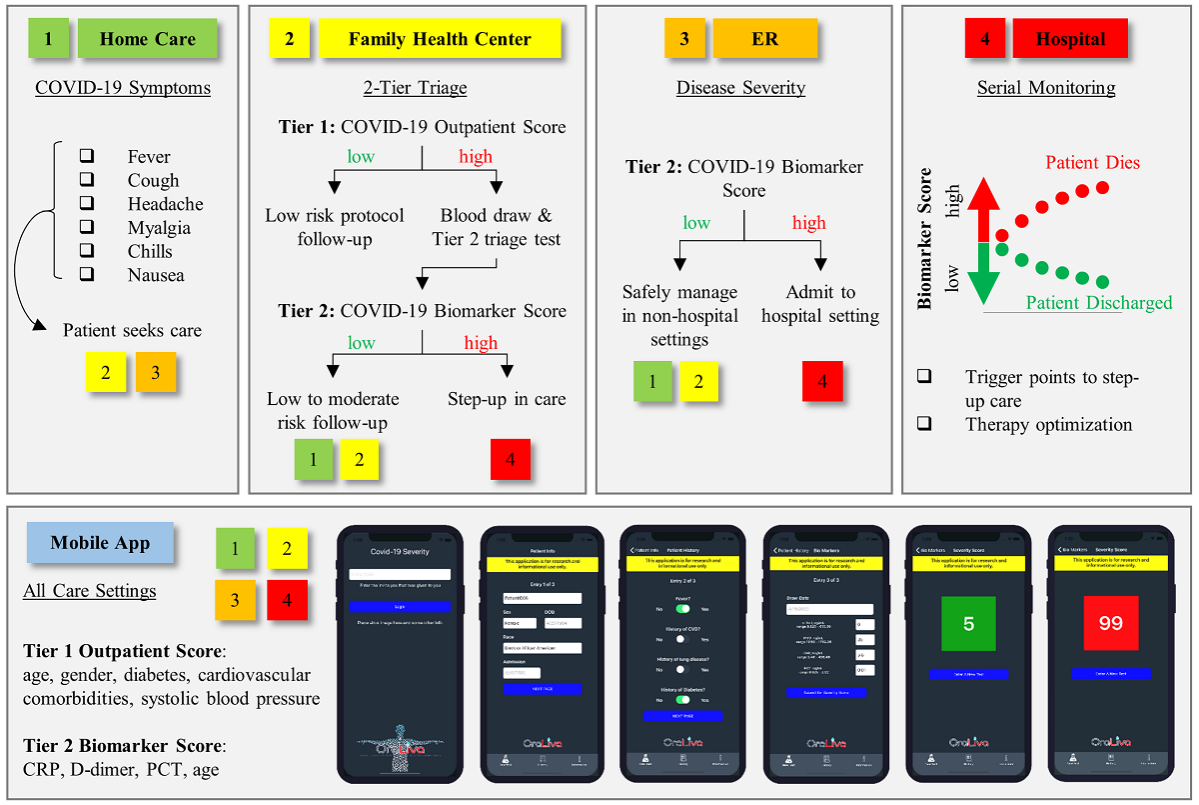
Clinical decision support system and mobile app for managing COVID-19 care. COVID-19: coronavirus disease; CRP: C-reactive protein; PCT: procalcitonin.

The CDSS and mobile app are designed to support decisions made in multiple settings, including home care, primary care or urgent care clinics, emergency departments, and hospital and intensive care (Figure 1). The process starts with symptomatic patients who are positive or presumably positive for COVID-19 and seeking care at a family health center or emergency room. In the family health center, decisions are made in two key stages, or tiers. The Tier 1 algorithm is intended for individuals in an outpatient setting where laboratory data are not yet readily available, and it uses only age, gender, blood pressure, and comorbidities. Patients with a low COVID-19 Outpatient Score may be managed in a home or telemedicine setting, while patients with a high COVID-19 Outpatient Score are referred for a blood draw and Tier 2 biomarker-based test. The Tier 2 algorithm, which is directly related to mortality risk, predicts disease severity using biomarker measurements and age. Patients with a low COVID-19 Biomarker Score are expected to be managed in a low-to-moderate risk group (eg, 5-day telehealth follow-up), while patients with a high COVID-19 Biomarker Score are expected to be hospitalized in most cases or managed in a high risk group (eg, 24- to 48-hour follow-up). Providers encountering clinically evident severe cases, as in urgent care or emergency departments, may choose to bypass the Tier 1 Outpatient Score and perform biomarker testing and Tier 2 triage on all patients with COVID-19. Last, in the hospital setting, patients are serially monitored for their COVID-19 Biomarker Scores. This personalized time series information directly related to mortality risk has strong potential to optimize therapy, improve patient care, and ultimately save lives. For both algorithms, we selected cutoffs that balanced sensitivity and specificity; however, these algorithms can be easily tuned for high sensitivity or high specificity by adjusting the weighting or relative importance of sensitivity and specificity in clinical practice.

Of the 701 patients with detectable COVID-19 infection cared for in one of the 9 clinics within the FHC network, 402 (57.3%) were not hospitalized, 185 (26.4%) were hospitalized and discharged, 19 (2.7%) were ventilated, and 95 (13.6%) died (Table 1). Ventilated and deceased patients were older than those who were not hospitalized or discharged (*P*=.03 and *P*<.001, respectively). Of patients who were ventilated and deceased, 14/19 (73.7%) and 60/95 (63.2%) were male, respectively, vs 271/587 (46.1%) for patients with less severe disease (ie, not hospitalized or discharged) (*P*=.02 and *P=*.002, respectively). Diabetes was also a statistically significant factor, with 9/19 (47.4%) and 52/95 (54.7%) in the ventilated and deceased groups vs 149/587 (25.3%) in the nonhospitalized and discharged groups (*P*=.03 and *P*<.001, respectively). Likewise, 10/19 (52.6%) of ventilated patients (*P=*.04) and 65/95 (68.4%) of deceased patients (*P*<.001) had one or more cardiovascular comorbidities, vs 181/587 (30.8%) for the less severe disease categories, with hypertension being the most common comorbidity. Interestingly, systolic blood pressure was significantly higher for patients who were not hospitalized vs those who were discharged (*P*=.004), and patients who died had abnormally low blood pressure relative to patients with less severe disease (*P*<.001). All biomarkers (cTnI, CRP, PCT, D-dimer, and NT-proBNP) were measured at significantly higher levels in patients who died vs those who were not hospitalized or discharged (*P*<.001).

**Table 1:** Characteristics of the patients included in model training. Data are represented as n (%), mean ± standard deviation, or median (IQR).

Characteristic	Not hospitalized (n=402)	Discharged (n=185)		Ventilated (n=19)			Deceased (n=95)	
	Value	Value	*P* value^a^	Value	*P* value^b^	Value		*P* value^b^
Age (years), mean (SD)	48 (17)	50 (17)	.32	58 (20)	.03	67 (14)		<.001
Male sex, n (%)	182 (45.3)	89 (48.1)	.52	14 (73.7)	.02	60 (63.2)		.002
BMI, kg/m^2^, mean (SD)	25 (4)	28 (6)	.16	29 (5)	.46	25 (6)		.06
Systolic BP^c^ (mm Hg), mean (SD)	132 (14)	123 (19)	.004	126 (20)	.78	94 (40)		<.001
Diastolic BP (mm Hg), mean (SD)	82 (8)	71 (11)	<.001	70 (12)	.29	54 (26)		<.001
Temperature (ºF), mean (SD)	99 (1)	98 (5)	.54	99 (1)	.66	100 (2)		.12
Pulse (beats per minute), mean (SD)	90 (18)	84 (14)	.06	93 (14)	.03	74 (54)		.02
Asthma, n (%)	44 (10.9)	12 (6.5)	.09	3 (15.8)	.37	6 (6.3)		.31
COPD^d^, n (%)	60 (14.9)	17 (9.2)	.06	3 (15.8)	.74	15 (15.8)		.48
Cancer, n (%)	13 (3.2)	5 (2.7)	.73	2 (10.5)	.07	14 (14.7)		<.001
Cardiovascular comorbidities^e^, n (%)	120 (29.9)	61 (33.0)	.45	10 (52.6)	.04	65 (68.4)		<.001
Diabetes, n (%)	96 (23.9)	53 (28.6)	.22	9 (47.4)	.03	52 (54.7)		<.001
HIV/AIDS, n (%)	3 (0.7)	2 (1.1)	.68	0 (0.0)	.69	3 (3.2)		.053
Liver disease, n (%)	11 (2.7)	10 (5.4)	.11	2 (10.5)	.12	4 (4.2)		.76
Renal disease, n (%)	20 (4.9)	17 (9.2)	.051	3 (15.8)	.10	21 (22.1)		<.001
cTnI^f^ (pg/mL), median (IQR)	7.07 (7.07-7.07)	7.07 (7.07-7.07)	.30	20.00 (7.07-63.75)	<.001	73.50 (7.07-712.00)		<.001
CRP^g^ (mg/L), median (IQR)	51.40 (16.55-101.35)	67.90 (17.95-121.50)	.28	37.30 (27.30-139.72)	.44	176.00 (115.00-287.00)		<.001
PCT^h^ (ng/mL), median (IQR)	0.12 (0.06-0.36)	0.10 (0.05-0.31)	.31	0.69 (0.07-1.91)	.008	1.61 (0.35-8.31)		<.001
D-Dimer (μg/mL^i^), median (IQR)	0.39 (0.20-0.71)	0.27 (0.18-0.56)	.047	0.86 (0.50-3.02)	<.001	1.58 (0.72-5.35)		<.001
NT-proBNP^j^ (pg/mL), median (IQR)	93.00 (36.50-375.25)	88.00 (28.50-298.00)	.60	217.00 (78.00-394.25)	.13	937.00 (160.25-5728.50)		<.001

^a^Compared to patients who were not hospitalized.

^b^Compared to patients who were not hospitalized or discharged.

^c^BP: blood pressure.

^d^COPD: chronic obstructive pulmonary disease.

^e^Cardiovascular comorbidities: one or more of cerebrovascular disease, heart failure, ischemic heart disease, myocardial infarction, peripheral vascular disease, and hypertension.

^f^cTnI: cardiac troponin I.

^g^CRP: C-reactive protein.

^h^PCT: procalcitonin.

^i^µg/mL: micrograms per milliliter.

^j^NT-proBNP: N-terminal fragment of the prohormone brain natriuretic peptide.

### Tier 1 Outpatient Model

**Figure 2:**
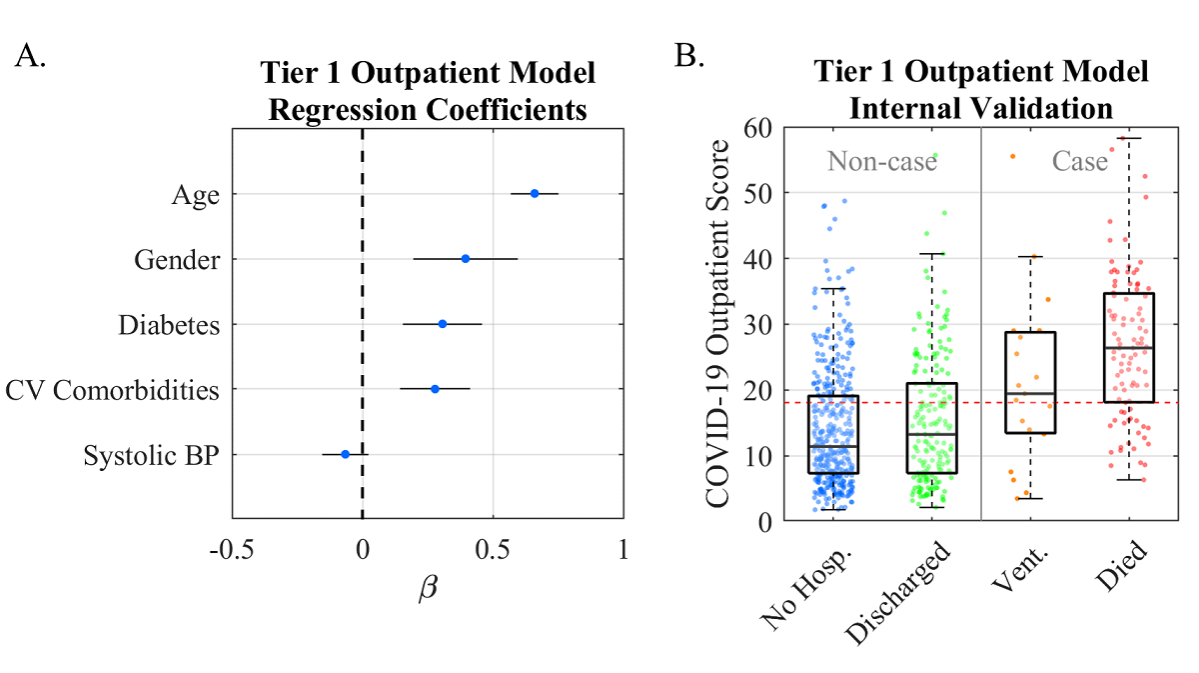
Validation of the Tier 1 Outpatient Model. A. Lasso logistic regression coefficients revealing the relative importance of predictors in generating the score. B. Box/scatter plot from the internal validation showing the Tier 1 Outpatient Scores for the four outcomes. A cutoff score of 18 (red dotted line) balances sensitivity and specificity for “Noncase” vs “Case” patients (gray line). COVID-19: coronavirus disease; CV comorbidities: cardiovascular comorbid conditions; No Hosp.: patients who were not hospitalized; Vent.: patients who were ventilated.

The Tier 1 Outpatient Model for COVID-19 disease severity was developed and internally validated using data from the FHCs at NYU Langone (Figure 2). The model retained the following predictors: age, gender, systolic blood pressure, cardiovascular comorbidities (one or more of cerebrovascular disease, heart failure, ischemic heart disease, myocardial infarction, peripheral vascular disease, and hypertension), and diabetes. The median COVID-19 Outpatient Scores were 11, 13, 20, and 27 for not hospitalized, discharged, ventilated, and deceased patients, respectively. The AUC of the model was 0.79 (95% CI 0.74-0.84) at the optimal cutoff COVID-19 Outpatient Score of 18 (Table 2). The median scores (Figure 2) had statistically significant differences for comparisons between all patient groups, except for not hospitalized vs discharged (*P*=.18).

**Table 2:** Internal validation performance in terms of AUC, sensitivity, specificity, PPV, and NPV (95% CI) from 5-fold cross-validation. The Tier 1 and 2 models were trained and tested using data from Family Health Centers at New York University.

	Tier 1 Outpatient Model	Tier 2 Biomarker Model
AUC^a^	0.79 (0.74-0.84)	0.95 (0.92-0.98)
Sensitivity	0.73 (0.69-0.76)	0.89 (0.86-0.92)
Specificity	0.73 (0.69-0.76)	0.89 (0.86-0.92)
PPV^b^	0.34 (0.30-0.38)	0.70 (0.65-0.74)
NPV^c^	0.93 (0.91-0.95)	0.97 (0.94-0.98)

^a^AUC: area under the curve.

^b^PPV: positive predictive value.

^c^NPV: negative predictive value.

### Tier 2 Biomarker Model

**Figure 3:**
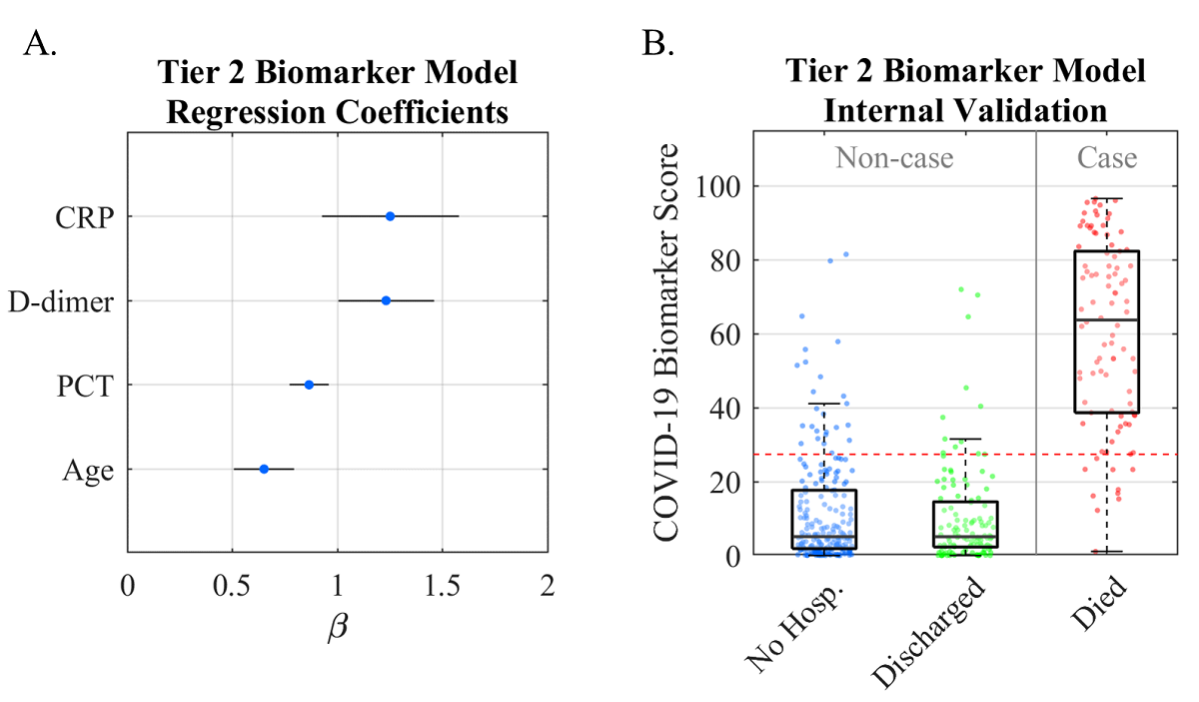
Validation of the Tier 2 Biomarker Model. A. Lasso logistic regression coefficients revealing the relative importance of predictors in generating the score. B. The box/scatter plot from internal validation shows Tier 2 Biomarker Scores for the three patient outcomes. A cutoff score of 27 (horizontal red dotted line) balances sensitivity and specificity for “Noncase” vs “Case” patients (vertical gray line) COVID-19: coronavirus disease; No Hosp.: patients who were not hospitalized.

The Tier 2 Biomarker Model for COVID-19 disease severity was developed and internally validated using data from the FHCs at NYU Langone (Figure 3). Patients who were ventilated (n=19) and still hospitalized (n=19) were excluded. Patients with fewer than one biomarker measurement were excluded (n=190 not hospitalized, n=64 discharged, n=1 deceased). The remaining 427 patients with one or more biomarker measurements were included in the analysis (n=212 not hospitalized, n=121 discharged, n=94 deceased). The model retained the following predictors after shrinkage and selection: age, D-dimer, PCT, and CRP. The median COVID-19 Outpatient Scores were 5, 5, and 64 for not hospitalized, discharged, and deceased patients, respectively. The AUC of the model was 0.95 (95% CI 0.92-0.98) at the optimal cutoff COVID-19 Outpatient Score of 27 (Table 2). The median COVID-19 Outpatient Scores (Figure 3) had statistically significant differences for comparisons between patients who were not hospitalized and patients who died (*P*<.001) and between patients who were discharged and patients who died (*P*<.001).

### External Validation

**Figure 4:**
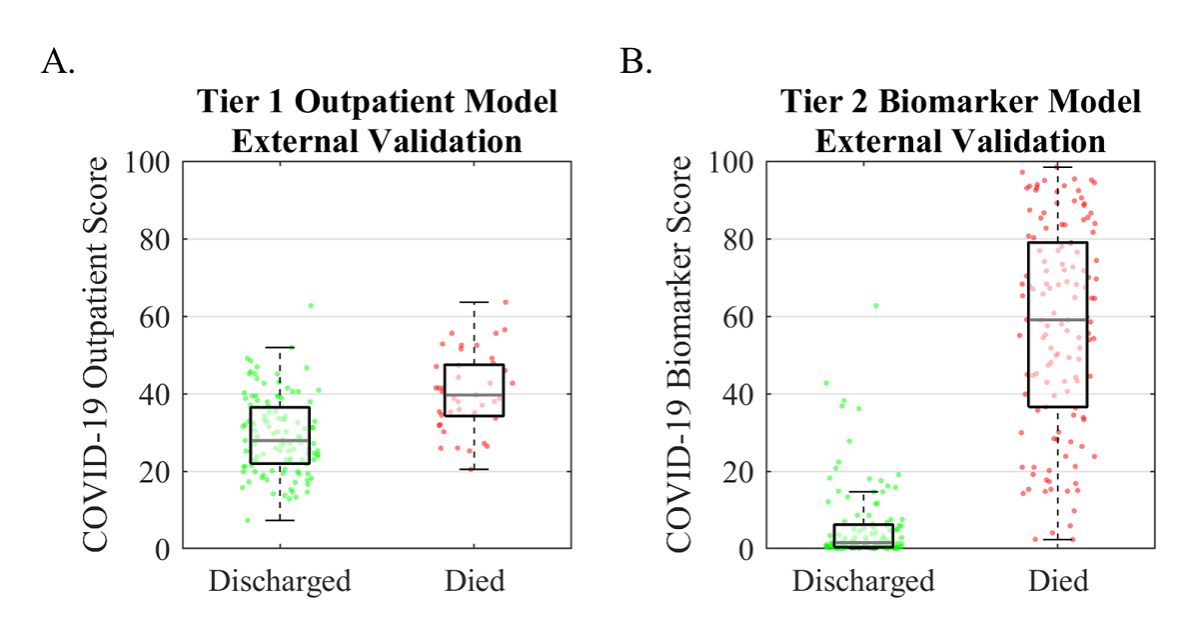
External validation results. A. The Tier 1 Outpatient Model was evaluated using data from patients with COVID-19 at Zhongnan Hospital of Wuhan University [26]. B. The Tier 2 Biomarker Model was evaluated using data from patients with COVID-19 at Tongji Hospital [21]. COVID-19: coronavirus disease.

We externally validated the Tier 1 Outpatient Model using data from a study of 160 hospitalized patients with COVID-19 who had hypertension from Zhongnan Hospital of Wuhan University, Wuhan, China [26]. Of the 160 patients in the study, 4 (2.5%) were missing one or more predictors and were excluded from the analysis. The COVID-19 Biomarker Scores were evaluated for 115 patients who were discharged and 41 patients who died (Figure 4A). The median COVID-19 Biomarker Scores were 27.9 (IQR 22.0-36.4) for patients who were discharged and 39.7 (34.2-47.4) for patients who died. The external validation diagnostic performance was determined using a cutoff score of 34 (Table 3).

We externally validated the Tier 2 Biomarker Model using data from a study of 375 hospitalized COVID-19 patients from Tongji Hospital in Wuhan, China, collected between January 10 and February 18, 2020 [21]. To maximize potential lead time, the first available laboratory measurements during hospitalization were used to generate cross-sectional COVID-19 Biomarker Scores, representing the first in a series of measurements collected for hospital stays lasting a median of 12.5 (IQR 8-17.5) days prior to the outcomes (discharged or deceased). Out of the 375 patients in the study, 133 were missing one or more lab values and excluded from the analysis. The COVID-19 Biomarker Scores were evaluated for 112 patients who were discharged and 130 patients who died (Figure 4B). The median COVID-19 Biomarker Scores were 1.6 (IQR 0.5-6.2) for patients who were discharged and 59.1 (IQR 36.6-78.9) for patients who died. The external validation diagnostic performance was determined using a cutoff score of 19 (Table 3).

**Table 3:** External validation performance in terms of AUC, sensitivity, specificity, PPV, and NPV (95% CI). The Tier 1 Outpatient Model was evaluated on the Zhongnan Hospital dataset [26]. The Tier 2 model was evaluated on the Tongji Hospital dataset [21].

	Tier 1 Outpatient Model	Tier 2 Biomarker Model
AUC^a^	0.79 (0.70-0.88)	0.97 (0.95-0.99)
Sensitivity	0.76 (0.68-0.82)	0.89 (0.84-0.93)
Specificity	0.73 (0.65-0.80)	0.93 (0.89-0.96)
PPV^b^	0.50 (0.42-0.58)	0.94 (0.90-0.96)
NPV^c^	0.89 (0.83-0.94)	0.88 (0.83-0.92)

^a^AUC: area under the curve.

^b^PPV: positive predictive value.

^c^NPV: negative predictive value.

**Figure 5:**
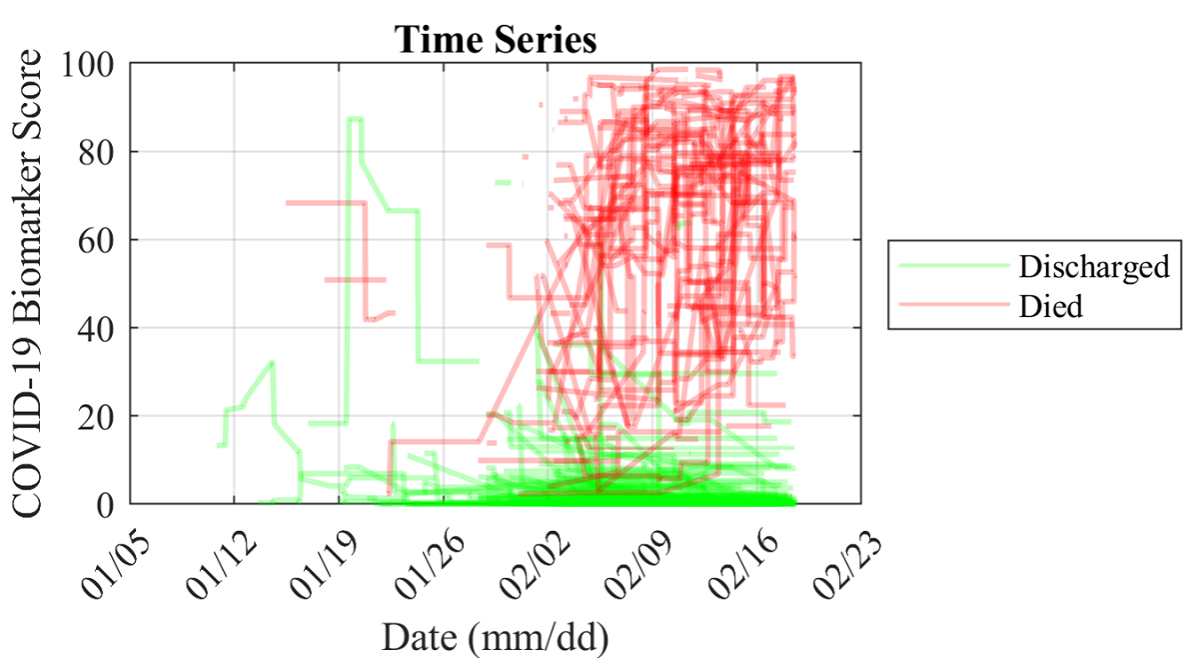
Spaghetti plot of longitudinal COVID-19 Biomarker Scores for patients in the external validation set from Tongji Hospital [21] between January 10 and February 18, 2020. These data represent individual patients’ scores over a median (IQR) of 12.5 (8–17.5) days between admission and outcomes of discharged or deceased. The first scores available after admission were significantly higher in those that died vs those that were discharged (AUC 0.97, cutoff score of 19), and over time patients who were discharged had an average decrease in score (-4.7) while those that died had an average increase in score (+11.2).

We also evaluated the COVID-19 Biomarker Scores for patients over time using longitudinal biomarker measurement data from individual patients in the external validation set (Figure 5). These data represent individual patients’ scores over a median of 12.5 days (IQR 8-17.5) between admission and outcomes of discharge or death. The first scores available after admission were significantly higher in patients who died vs patients who were discharged (AUC 0.97, cutoff score of 19); over time, patients who were discharged had an average decrease in score (–4.7), while patients who died had an average increase in score (+11.2).

## Discussion

As the COVID-19 pandemic continues to create surges and resurgences without an effective vaccine, the goal of this multidisciplinary team was to develop a triage and prognostication tool that strengthens community-level testing and disease severity monitoring. A CDSS and mobile app for COVID-19 severity have been designed, developed, and validated using data from 1236 patients with COVID-19 across numerous clinics and hospitals in the coronavirus disease epicenters of Wuhan, China, and New York, United States. These clinically validated tools have potential to assist health care providers in making evidence-based decisions in managing the care of patients with COVID-19. The significance of this work is realized by the algorithms developed and validated here, which are accurate, interpretable, and generalizable.

Accurately identifying patients with elevated risk for developing severe COVID-19 complications can empower health care providers to save lives by prioritizing critical care, medical resources, and therapies. With respect to accuracy, both Tier 1 and Tier 2 models were effective in discriminating disease outcomes, with statistically significant differences between the most relevant patient groups (AUCs of 0.79 and 0.97 for Tier 1 and Tier 2 external validation, respectively). As expected, the diagnostic accuracy of the Tier 1 Outpatient Model in terms of AUC was lower than that of the Tier 2 Biomarker Model, which demonstrates the importance of biomarker data in determining disease severity. The accuracy with which the Tier 2 Biomarker Score identified patients who eventually died reflects the unfortunate and morbid reality of the COVID-19 pandemic to date. However, as medical knowledge and experience with COVID-19 progresses, it is possible that future treatments and interventions could improve patient survival. In this context, the Tier 2 Biomarker Score could be used to monitor patients’ treatment progression or regression over time and modify therapies accordingly.

Another strength of this approach is the interpretability of the models. While many predictive tools rely on “black box” methods in which algorithmic decisions and the logic supporting those decisions are uninterpretable, the lasso logistic regression method is transparent through its coefficients (ie, log odds) and probabilistic output. The Tier 1 Outpatient Score is the probability of severe disease (ventilation or death) based on the predictors (age, gender, diabetes, cardiovascular comorbidities, and systolic blood pressure). Likewise, the Tier 2 Biomarker Score is the probability of mortality based on CRP, D-dimer, PCT, and age. Predictive models such as these are more likely to be adopted for clinical applications in which transparency and interpretability are valued.

One of the most clinically relevant features of this new CDSS is the capacity to monitor individual patients over time. The use of this *precision diagnostic* approach allows for the amplification of early signs of disease, which can be achieved by focusing on time-course changes of biomarker signatures that are referenced not to population metrics, but rather back to the individual patient. As an example, the use of time course changes in individual biomarker fingerprints has been explored previously in the study of early detection in ovarian cancer [27]. Studies demonstrated that cancer antigen 125 by itself for a single time point was a poor diagnostic marker due to overlapping reference range problems across the population. However, when each patient was treated as their own point of reference and biomarker slopes for individual patients were considered, the diagnostic accuracy for this same biomarker increased significantly. Similarly, the COVID-19 Biomarker Score time series (Figure 5) reveals a strong capacity to separate patients who die of COVID-19 complications from those who are discharged from the hospital. Note that the app includes capabilities to use proximal biomarker measurements, allowing for biomarker measurements to be collected over time without the rigid restriction of requiring completion of all biomarker measurements at the same time for all time points. This flexibility is anticipated to afford more convenience for longitudinal monitoring of patients.

Lastly, the models developed here demonstrated generalizability through external model validation. External validation is essential before implementing prediction models in clinical practice [28]. We found that the AUCs for both the Tier 1 and Tier 2 models were similar for internal vs external validation, demonstrating that the models are generalizable to making predictions for these disease indications in different care settings and for different patient demographics. Usually, prediction models perform better on the training data than on new data; however, in this study, we found that the external validation results were approximately the same or better (Tier 1: AUC of 0.79 vs 0.79; Tier 2: 0.95 and 0.97 for internal and external validation, respectively), suggesting that patients in the external validation sets may have suffered from more severe disease.

Despite the potential for CDSSs to transform health care, major challenges remain for translating and scaling these tools. Future data and, thus, future model performance may have large heterogeneity, which may be exacerbated by missing data (potentially not missing at random), nonstandard definitions of outcomes, and incomplete laboratory measurements and follow-up times [29]. The mobile app developed here is intended to reduce heterogeneity by encouraging the harmonization of data collection across multiple care settings. Further, models may be tuned through optimization of cutoffs for certain patient subpopulations. Another challenge in deploying a CDSS that relies on biomarker measurements is accounting for differences in laboratory testing across hospitals and clinics. The variability of these measurements across institutions may have a large impact on the distribution of COVID-19 Biomarker Scores. This challenge creates a unique opportunity for standardized, well-calibrated, and highly scalable point-of-care tests for COVID-19 disease severity [20,30,31]. Finally, the COVID-19 pandemic is a fluid and rapidly evolving crisis. Not only will our epidemiological and physiological understanding of the disease evolve over time, but viral mutations could also alter disease severity in future outbreaks. The two-tiered algorithms developed here are highly amenable to future adaptations in which new data are included in the training through periodic or continuous learning.

A commercial app has been developed in collaboration with OraLiva, Inc for deployment of these tools to frontline health care workers managing COVID-19 patients. Plans are now in place to assess the usability, user satisfaction, and confidence in results of this CDSS and mobile app in the FHCs at NYU. Future efforts will focus on point-of-care testing capabilities to more rapidly assess the Tier 2 biomarkers described in this study using a previously developed and published platform [20,30,31]. The deployment of these new capabilities has potential for immediate clinical impact in community clinics, where the application of these tools could significantly improve the quality of care.

## Abbreviations

APACHE: Acute Physiology and Chronic Health Evaluation
AUC: area under the curve
CDSS: clinical decision support system
COVID-19: coronavirus disease
CRP: C-reactive protein
cTnI: cardiac troponin I
eICU: electronic intensive care unit
FHC: Family Health Centers
ICU: intensive care unit
NPV: negative predictive value
NT-proBNP: N-terminal fragment of the prohormone brain natriuretic peptide
NYU: New York University
PCT: procalcitonin
PPV: positive predictive value
SARS-CoV-2: severe acute respiratory syndrome coronavirus 2
SOFA: Sepsis-related Organ Failure Assessment
